# Determination of Saffron Quality through a Multi-Analytical Approach

**DOI:** 10.3390/foods11203227

**Published:** 2022-10-15

**Authors:** Andrea Bergomi, Valeria Comite, Laura Santagostini, Vittoria Guglielmi, Paola Fermo

**Affiliations:** Department of Chemistry, University of Milan, Via Golgi 19, 20133 Milano, Italy

**Keywords:** saffron, UV-vis spectroscopy, SEM-EDX, ATR-FTIR, ICP-OES, food quality

## Abstract

Currently, the specifications for the assessment of saffron quality are contained in the ISO 3632 technical standard. This norm evaluates saffron quality through a UV-Vis spectrophotometric method and grades the spice into three commercial categories. However, numerous studies have highlighted several weaknesses and limitations of the ISO method. For this reason, a new multi-analytical approach for the determination of saffron quality is proposed in this work. Different techniques were employed to assess saffron quality: UV-visible spectroscopy (UV-Vis), attenuated total reflectance Fourier transform infrared spectroscopy (ATR-FTIR), scanning electron microscopy coupled with energy dispersive X-ray spectroscopy (SEM-EDX) and inductively coupled plasma—optical emission spectroscopy (ICP-OES). The results show that the commercial grading based on the ISO 3632 methodology is not always in line with the observations made with the other techniques. Moreover, the use of two new techniques in the field of saffron quality determination, i.e., SEM-EDX and ICP-OES, proved to be effective for the determination of elemental composition and metal content, which are two important parameters to take into consideration when assessing the quality of the spice.

## 1. Introduction

*Crocus sativus* L., commonly known as saffron, is the most cultivated plant species belonging to the *Crocus* genus [1]. It grows predominantly on the highlands and mountain areas of Western Asia and Mediterranean Europe thanks to the optimal continental-temperate or continental-Mediterranean climates [2]. Iran is the main producer with over 160 tons/year, representing around 90% of the total production, whereas other producers include India, Greece, Italy and Spain [3]. One of saffron’s unique features is represented by the three stigmas growing from the flower, which are responsible for the characteristic red colour of the final product; they are manually or mechanically separated and dried to produce commercially available saffron in filaments or powder [4].

Saffron is mainly used as a spice added to numerous foods and drinks [5], as well as a key component of many dyes used in the cosmetic industry [6]. Saffron is also a strong antioxidant and anti-inflammatory agent with several therapeutic properties [7]. For centuries, saffron extracts have been used to treat numerous conditions such as genital diseases, eye diseases and menstrual disorders [8]. More recently, new pharmacological effects of saffron and its metabolites have been discovered, including anti-depressant effects [9], improvement of sleep quality [10], effects in aiding depression and anxiety [11] and many others [8].

Due to a series of reasons, such as a long and laborious process necessary to achieve the final product [12] and the need for between 22,000 and 300,000 flowers to produce 1 kg of stigmas [13], saffron is the highest priced high value agricultural product (HVAP), reaching up to 20,000 EUR/kg [14]. Therefore, this spice is a major candidate for adulteration, especially the product sold as powder, in which extraneous material can be more easily introduced [13,15]. Throughout history, examples of adulteration involved mixing with condensed or older saffron and the addition of artificial substances, organic dyes, other parts of the saffron plant, substances that increase weight such as syrup, honey or glycerine, parts from other plants, or animal substances [16]. Hence, proper control of the quality of saffron is an important issue concerning the food industry as well as consumers. 

Currently, the norm assessing the quality of saffron and specifying the methods to detect adulteration is ISO 3632, which is divided in two parts: ISO 3632-1:2011 (Specifications) and ISO 3632-2:2010 (Test Methods) [17,18]. The latter describes a UV-Visible (UV-Vis) spectrophotometric method to classify saffron into three commercial categories (as reported in Table 1) based on the absorbance of a 1% solution by weight at three different wavelengths (440 nm, 270 nm and 330 nm), which are correlated, respectively, to the content of crocin, picrocrocin and safranal. Crocin is the main carotenoid of saffron and is responsible for the colour of the spice, picrocrocin is the molecule which is responsible for its bitter taste, whereas safranal is one of the many molecules imparting the characteristic aroma [8]. For these reasons, the absorbances previously mentioned are commonly referred to as: “colouring power”, “bittering power” and “odorous power” of saffron. 

In order to be classified as category I, II or III, the sample needs to satisfy the requirements of all three parameters and have a moisture and volatile matter content below 12% for saffron filaments or below 10% for saffron powders [18]. 

Despite the fact that the ISO 3632 grading remains the main frame of reference for saffron quality, several studies suggest that the spectrophotometric method is not the best way to assess the overall quality of this spice [19]. One of the main limitations is the inability to correctly quantify the amount of safranal in the sample [19,20,21] and consequently the failure to assign saffron to the proper commercial category. Despite attempts made to quantify the three molecules responsible for saffron quality through the use of an alternative HPLC-DAD methodology [20], other studies show that the problems of the ISO method do not lie solely in the detection of safranal [3,14,22,23]. For example, the method is incapable, in certain cases, of revealing adulterations with other types of plants, and the use of diffuse reflectance infrared Fourier transform spectroscopy (DRIFTS) needs to be employed as an alternative [15]. Moreover, the ISO method is also unable to distinguish between synthetic components and natural ingredients; in this case a three-step approach focused on the use of two innovative techniques based on microscopic examinations and DNA barcoding needs to be performed in addition to the ISO methodology [3]. 

An exhaustive review on the methods found in the literature that have been used for the determination of saffron quality shows that, aside from the ISO methodology, quality assessment has been performed using chromatographic, spectroscopic and biomimetic-based techniques, along with molecular-biological methods [22]. One of the main points of agreement between the cited methods is that every technique can give valuable information regarding the quality of the spice; nonetheless, no technique is fully exhaustive in performing this evaluation, suggesting that saffron quality can be determined only by using an array of techniques. 

In this study, a new multi-analytical approach for the determination of saffron quality is presented, based on the use of two widespread and consolidated techniques, i.e., UV-Vis spectrophotometry (as described in the ISO 3632 standard) and attenuated total reflectance Fourier transform infrared (ATR-FTIR) spectroscopy, along with two novel techniques in the field of saffron quality determination, which are scanning electron microscopy coupled to energy dispersive X-ray spectroscopy (SEM-EDX) and inductively coupled plasma optical emission spectroscopy (ICP-OES). 

Despite its several weaknesses, the ISO methodology remains a point of reference when performing analyses on saffron samples, and therefore has been included as part of our study. ATR-FTIR spectroscopy is a rapid screening technique requiring minimal sample preparation that provides a characteristic fingerprint of the spice [24], which is crucial in sample authentication. Moving on to the new techniques, SEM-EDX is a simple and fast technique which is used to perform morphological investigations and, thanks to the EDX probe, also the determination of the elemental composition of the samples. ICP-OES is employed for the quantification of several metals due to its good sensitivity and large applicability. Thanks to the results obtained with the use of these two techniques, elemental composition and metal content are proposed in this work as two new parameters that can be considered when assessing saffron quality. 

## 2. Materials and Methods

A total of 21 saffron samples were examined; 9 in powder form and 12 in filaments (Table 2). Both private and commercial samples of various origins were analyzed. The latter definition refers to samples which have been packaged, labelled and were available on the market for purchase, whereas the former refers to samples which have been harvested and processed by private collectors. Prior to analysis, all of the samples were stored at 4 °C in the absence of light. 

### 2.1. Determination of Moisture and Volatile Matter Content

Saffron filament samples were crushed using a pestle in an agate mortar until reaching a fine powder, whereas saffron powder samples required no pretreatment. Around 1.000 g of both saffron powder and saffron filaments was weighed to the nearest 0.001 g. The samples were then oven-dried at 103 ± 2 °C for 4 h and allowed to cool in a desiccator. Once they reached room temperature, they were weighed to the nearest 0.001 g. 

Results were expressed as percentage of moisture and volatile matter content according to the following equation:

(1) $$M_{VOL}\left(\%\right)=100-\left(\frac{m_{f}}{m_{i}}\times 100\right).$$

In this equation, m_i_ is mass of the sample prior to drying and m_f_ is the mass after drying. 

### 2.2. UV-Vis Spectroscopy

The analyses were carried out following the specifications indicated in Paragraph 14 of the ISO 3632-2:2010 norm with a UVIKON 943 double-beam UV-Vis spectrophotometer (Kontron Instruments, Milan, Italy). Around 125 mg of dried saffron was transferred to a 250 mL volumetric flask and approximately 230 mL of Milli-Q water was added. The flask was covered with aluminum foil to avoid direct contact with light sources, placed in a cold-water bath (<22 °C), and the content was stirred for 1 h. The solution was diluted up to the mark with Milli-Q water, then 60 mL was filtered using rapid filtration filter paper (Extra Rapida—Perfecte 2, myCordenons, Milan, Italy). The first 40 mL was discarded and the last 20 mL was kept. Half of this volume was withdrawn and diluted up to the mark in a 100 mL volumetric flask.

The absorption characteristics of this solution were measured by recording a spectrum between 200 nm and 700 nm. Maximum absorbances at 440 nm, 257 nm and 330 nm were recorded, corresponding to the absorption of crocin, picrocrocin and safranal, respectively. The results were expressed as the “coloring power”, “bittering power” and “odorous power” of saffron using the following equations:

(2) $$\mathrm{Colouring}\;\mathrm{power}=A_{1\;\mathrm{cm}}^{1\%}\left(440\;\mathrm{nm}\right)=\frac{A_{440\;\mathrm{nm}}\times 2500}{m_{w}\times W_{d}\left(\%\right)},$$

(3) $$\mathrm{Bittering}\;\mathrm{power}=A_{1\;\mathrm{cm}}^{1\%}\left(257\;\mathrm{nm}\right)=\frac{A_{257\;\mathrm{nm}}\times 2500}{m_{w}\times W_{d}\left(\%\right)},$$

(4) $$\mathrm{Odorous}\;\mathrm{power}=A_{1\;\mathrm{cm}}^{1\%}\left(330\mathrm{nm}\right)=\frac{A_{330\;\mathrm{nm}}\times 2500}{m_{w}\times W_{d}\left(\%\right)},$$

where A_λ,max_ is the maximum absorbance at the desired wavelength, m_w_ is the mass of the sample in grams, and W_d_(%) is the percentage weight of dry sample.

### 2.3. ATR-FTIR Spectroscopy 

A small portion of the sample was retrieved and placed under the tip of a Nicolet 380 FTIR spectrometer (Thermo Electron Corporation, Waltham, MA, USA). A spectrum between 400 cm^−1^ and 4000 cm^−1^ was recorded in transmittance mode using ATR as a sampling technique and carrying out 64 scans with a resolution of 4 cm^−1^ and performing smoothing operations (15 points). 

### 2.4. Scanning Electron Microscopy Energy Dispersive X-ray Spectroscopy (SEM-EDX)

An appropriate amount of powder or filaments was placed on a standard circular 19 mm pin stub previously covered with a graphite coating and analyzed using a TM4000PlusII Scanning Electron Microscope (Hitachi, Tokyo, Japan) coupled with an EDX microprobe. Nine different areas were selected (3.8 mm × 2.4 mm) with a 50× magnification and for each of them a microscopic image was obtained using back-scattered electrons in low vacuum conditions. Point and aerial analyses at greater magnifications were performed when necessary. Semi-quantitative elemental analysis of saffron’s main components was carried out with the EDX microprobe on all of the nine areas mentioned and mean average values were then calculated.

### 2.5. Principal Component Analysis (PCA)

Principal component analysis (PCA), which is an unsupervised method used to evaluate similarities and differences between samples in terms of elemental composition, was carried out on EDX data acquired as described in the previous section. Score plots and loading plots were obtained from the starting dataset and evaluated. All analyses were carried out with the statistical package STATISTICA 7.1 (StatSoft Italia, Vigonza, Italy).

### 2.6. Inductively Coupled Plasma Optical Emission Spectroscopy (ICP-OES) 

A total of 13 different metals were analyzed using an Optima 8000 Optical Emission Spectrometer (Perkin Elmer, Waltham, MA, USA): Na, Mg, Ca, K, Al, Fe, Pb, Cd, Cr, Mn, Zn, Ni and Cu. 

#### 2.6.1. Reagents

The reagents used in the digestion procedures were: nitric acid (67%, NORMATOM^®^, Ultrapure, VWR Chemicals, Radnor, PA, USA), perchloric acid (65–71%, NORMATOM^®^, Ultrapure, VWR Chemicals, Radnor, PA, USA) and hydrogen peroxide (30%, NORMATOM^®^, Ultrapure, VWR Chemicals, Radnor, PA, USA). High-purity Milli-Q water (Merck Millipore Milli-Q, Burlington, MA, USA) was employed in all the steps that required its use. 

Calibration curves were constructed using multielement external standards. These were prepared starting from 1000 mg L^−1^ single-element standard solutions (Perkin Elmer Pure, Atomic Spectroscopy Standard, Shelton, CT, USA). 

#### 2.6.2. Sample Preparation

Two different digestion procedures were carried out to perform the ICP-OES analysis on the samples: open vessel (OV) and microwave-assisted (MW) digestion. Both have been used in the determination of the metal content of saffron [25,26] and both possess qualities that make them suitable for this type of analysis [27].

With regards to the first, approximately 50 mg of dried saffron was weighed to the nearest 0.1 mg. A solution containing nitric acid and perchloric acid in a 10:1 ratio was prepared and 10 mL was transferred to a clean and dry Teflon container with the previously weighed saffron. The solution was heated at 120 °C on a heating plate for 2 h. 

Moving on to the second method, the same amount of saffron was weighed to perform the MW digestion and transferred in a PFA vessel along with 5 mL of nitric acid and 1 mL of hydrogen peroxide. The vessel underwent the following mineralization program (“W” stands for Watt, unit of measure of power):

1. 1 min—250 W
2. 1 min—0 W
3. 5 min—250 W
4. 4 min—400 W
5. 4 min—600 W

Both solutions were cooled at room temperature and diluted with Milli-Q water to a final volume of 25 mL. They were then filtered using 0.45 μm non-sterile hydrophilic membranes (PTFE Millex-14 LCR, 25 mm, Millex® Syringe Filters, Merck Millipore, Burlington, MA, USA) and centrifuged at 3000 rpm for 3 min. 

#### 2.6.3. Calibration

A series of standard solutions between 0.005 and 1 mg L^−1^ were prepared for each metal and analyzed. Calibration curves were constructed, and regression analysis performed: correlation coefficients were no less than 0.98. The values for the limit of detection (LOD) and limit of quantification (LOQ) for each metal were calculated using the following formulas:

(5) $$LOD=\frac{S_{b}+3s_{b}}{m}$$

(6) $$LOQ=\frac{S_{b}+10s_{b}}{m}$$

where *S_b_* is the average blank signal, *s_b_* is the blank standard deviation and m the slope of the calibration curve. LOD values ranged between 0.7 μg L^−1^ and 75 μg L^−1^, whereas LOQ values ranged between 2 μg L^−1^ and 90 μg L^−1^. 

## 3. Results

### 3.1. Determination of Moisture and Volatile Matter Content

The amount of moisture and volatile matter in saffron samples is an important parameter in defining the quality of the final product and values outside the recommended ranges are considered as signs of adulteration [13]. The percentage of moisture and volatile matter content ranged between 4.524% and 9.324% for powder samples and between 4.126% and 11.653% for saffron in filaments (Table 3). 

All of the values were below the thresholds indicated in the ISO 3632 standard (10% for powder samples, 12% for saffron in filaments) and no noteworthy differences can be observed between powder and filament samples. This was expected for commercial products, which are specifically treated in order to achieve values in the desired range, whereas samples obtained directly from producers often exceed the limits and can have moisture values above 30% [13]. 

### 3.2. UV-VIS Spectroscopy

**Figure 1:**
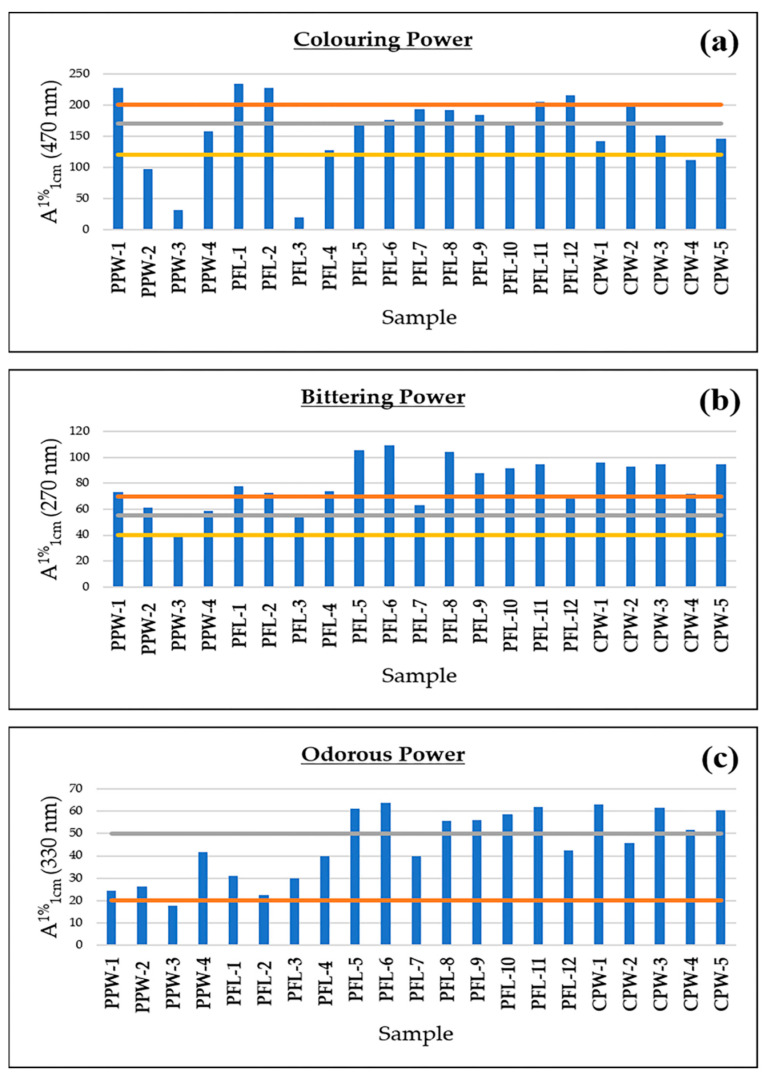
Bar graph showing the results for the coloring power (**a**), bittering power (**b**) and odorous power (**c**) of saffron. The horizontal lines indicate the thresholds of the different commercial categories indicated in the ISO 3632 norm.

Based on the results of the UV-VIS spectrophotometric analysis (Figure 1), four samples (PPW-1, PFL-1, PFL-2, PFL-12) were placed in category I, two samples (CPW-2, PFL-7) in category II, and another two samples (PPW-4, PFL-4) in category III. All of the others had at least one out of the three parameters which did not meet the criteria needed for classification, therefore no category could be assigned to them and, according to the ISO 3632 standard, these are the samples of the lowest quality possible.

It is worth noting that, of the 13 samples that were denied classification in one of the three commercial categories, 9 (CPW-1, CPW-3, CPW-5, PFL-5, PFL-6, PFL-8, PFL-9, PFL-10, PFL-11) were excluded solely due to the odorous power being outside the specified range, whereas the colouring and bittering power were at least above the minimum value required for category III classification. Only four samples (CPW-4, PFL-3, PPW-2, PPW-3) were denied classification because of low colouring and/or bittering power values. 

### 3.3. ATR-FTIR Spectroscpy

**Figure 2:**
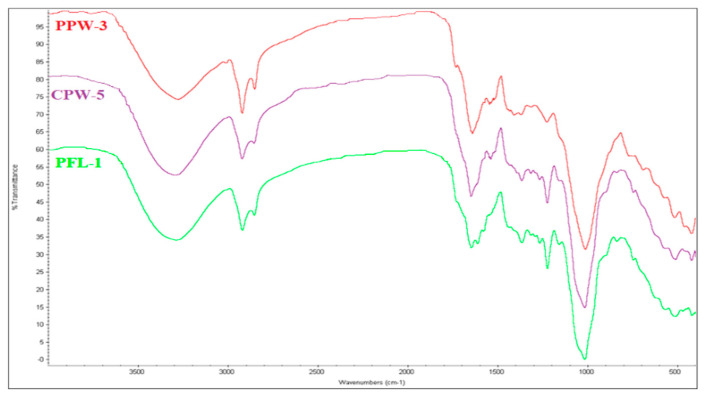
ATR-FTIR spectrum of samples PPW-3, CPW-5, PFL-1.

The FTIR spectra of all the samples show the same features of the reference FTIR spectrum of saffron [24]. This is shown in Figure 2, in which three FTIR spectra obtained on samples of different type and origin are represented. All of the major bands were present and correlated well with the reference spectrum in terms of relative height and position, starting from the broad band around 3350 cm^−1^ relative to the stretching vibrations of the -OH groups, moving on to the asymmetric and symmetric stretching vibrations of -CH_2_- and -CH_3_- groups at 2925 cm^−1^ and 2860 cm^−1^, the -C=O stretching vibrations around 1720 cm^−1^ and the C-4-OH glucose residue bending vibration at 1020 cm^−1^ [24]. 

Despite the fact that it is difficult to determine saffron quality exclusively by ATR-FTIR spectroscopy [28], the experimental spectra still represent a fingerprint for this type of spice [24]. In this light, it is safe to say that all of the samples analyzed were indeed composed of saffron and no major adulterations were present. Thanks to this authentication, it was possible to proceed with the final two analyses on all of the samples, which would otherwise have been insignificant. 

### 3.4. Scanning Electron Microscopy (SEM)

**Figure 3:**
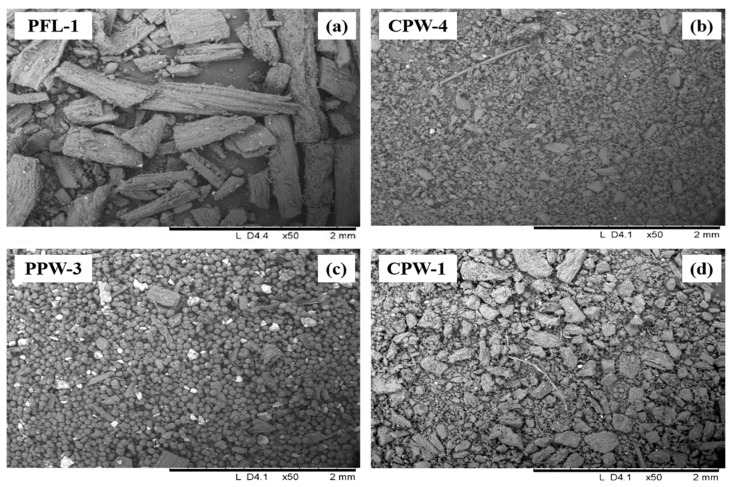
SEM images of samples (**a**) PFL-1; (**b**) CPW-4; (**c**) PPW-3; (**d**) CPW-1 (Area = 2.4 mm × 3.8 mm, 50× magnification).

Microscopic investigations on saffron filaments highlighted a homogenous composition between the different samples. The samples were composed mainly of irregular fragments greater than 1 cm in length and to a lesser extent of smaller jagged particles of irregular shape, probably deriving from the main filament (an example is shown in Figure 3a). No floral contaminants such as leaves or stamens were observed in any of these samples, confirming the fact that concealing these types of adulterants is more difficult if the product has not been crushed.

Saffron powders showed a much more heterogenous morphological appearance. Some of them (CPW-2, CPW-3, CPW-5, PPW-1) were composed almost exclusively of the same irregular fragments found in filament samples, only smaller in size (less than 1 cm in length). As in the case of the filament samples, no contaminants were detected in these powders. However, others showed a limited presence of these irregularly shaped fragments. In some cases, jagged particles of non-definable size and shape were more frequent (Figure 3b). In others it was possible to notice the presence of pollen granules (Figure 3c), which are spherical/ovoidal grains with an heterogenous size distribution [29]. Finally, in some cases it was possible to observe floral contaminants, such as in sample CPW-1 (Figure 3d).

These types of contamination arise from the cultivation and processing phases of saffron, highlighting lower quality operational practices or intentional contamination [4,16]. It is often the case that manual operations are substituted by semi-automatic machinery, which certainly speeds up the process, but inevitably gives a lower quality product contaminated with pollen granules [4].

**Figure 4:**
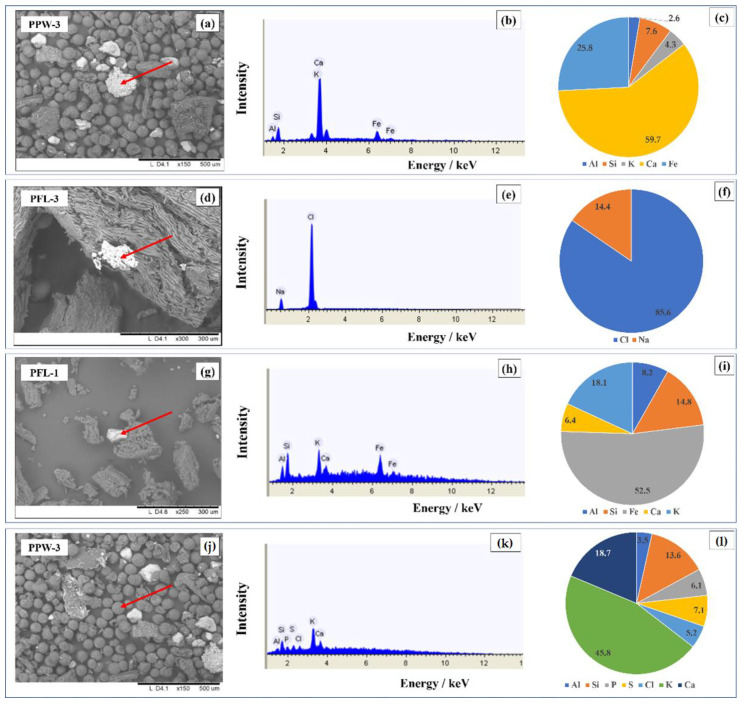
SEM-EDX analysis of the particles indicated by the red arrows. (**a**) SEM image of sample PPW-3 (1.1 mm × 0.87 mm, 150× magnification). (**b**) EDX spectrum relating to the analysis of the particle indicated in (**a**). (**c**) Elemental composition of the particle indicated in (**a**). (**d**) SEM image of sample PFL-3 (0.60 mm × 0.45 mm, 300× magnification). (**e**) EDX spectrum relating to the spectroscopic analysis of the particle indicated in (**d**). (**f**) Elemental composition of the particle indicated in (**d**). (**g**) SEM image of sample PFL-1 (0.60 mm × 0.45 mm, 300× magnification). (**h**) EDX spectrum relating to the spectroscopic analysis of the particle indicated in (**g**). (**i**) Elemental composition of the particle indicated in (**g**). (**j**) SEM image of sample PPW-3 (1.33 mm × 1.00 mm, 150× magnification). (**k**) EDX spectrum relating to the spectroscopic analysis of a pollen grain. (**l**) Elemental composition of the pollen grain.

Whereas floral contamination in commercial powder samples was only sporadic, samples PPW-2 and PPW-3 showed an extensive presence of pollen granules and almost none of the typical fragments previously observed. Moreover, as highlighted in Figure 3c, these samples were characterized by the widespread presence of brighter particles associated with heavier elements. EDX analyses of both the bright particles and the pollen granules highlighted the presence of terrigenous elements such as Al, Si, Ca, K and Fe (Figure 4a–c).

### 3.5. Energy Dispersive X-ray Spectroscopy (EDX)

Table 4 shows the elemental composition of the saffron samples analyzed, calculated as the average of nine aerial analysis (3.8 mm × 2.4 mm).

Being a fundamental plant nutrient crucial for plant growth [30], potassium was the element present in greater concentrations in all of the filament samples. Lower percentage concentrations were found in samples PFL-3 and PFL-4 mainly due to higher values of chlorine present in NaCl and KCl particles (Figure 4d–f). It is likely that this finding can be attributed to the presence of salt-rich aerosols, typical of maritime cities such as Genoa, where the samples are from.

Another important finding related to filament samples is that, despite the fact that Fe was not identified in any of the nine areas analyzed, punctual analysis revealed the presence of particles containing this element (Figure 4g–i). The elemental composition of the particles analyzed, as in the example in Figure 4i, shows a lower percentage of K when compared to the aerial analyses, but a higher concentration of Al and Si. This is likely due to impurities such as clay soil residues, dust or atmospheric particulate matter which came into contact with the sample during the cultivation or processing phases.

Saffron powders also showed K as the main constituent and lower concentrations were once again associated with a higher chlorine content (sample CPW-1). However, adulteration via addition of inorganic salts is suspected in this case, given that the origin of the sample cannot account for the higher chlorine content as in the filament samples.

Punctual analysis in all of the saffron powders revealed the presence of terrigenous particles such as the one presented in Figure 4a. In the case of commercial powders, no significant differences were observed in this regard when compared to filament samples. Instead, samples PPW-2 and PPW-3 showed a much greater presence of the aforementioned particles and this was also reflected in the higher average concentrations of Fe, Ca, Si and Al. These results are also due to the high number of pollen grains, which are rich in these elements (Figure 4j–l).

### 3.6. Inductively Coupled Plasma Optical Emission Spectroscopy

The results of the ICP-OES analysis conducted on samples PFL-1, PPW-3 and CPW-5 are reported in Table 5 and Table 6. Based on the results of the previous analyses, one sample of low (PPW-3), medium (CPW-5) and high (PFL-1) quality was chosen for this determination [27].

A clear trend can be observed between the different samples in terms of elemental concentration. Potassium was the most abundant element in all three samples and the one which showed the least variation, with values ranging between 5990 μg g^−1^ and 6840 μg g^−1^. Most of the other analytes (Mg, Al, Ca, Fe, Na) showed an increase in concentration moving from sample PFL-1 to CPW-5 and finally to PPW-3. The highest levels of Zn and Cu were also recorded for the latter. All of the other analytes (Cr, Mn, Pb, Ni, Cd) were found below the limit of quantification.

Considering that the elemental composition of saffron can vary greatly depending on numerous factors such as the origin of the sample [26], the values observed for samples PFL-1 and CPW-5 are comparable with literature data from other studies [25,26]. However, the results obtained for sample PPW-3 are systematically higher than the ones observed in other studies for all of the elements, with the exception of K.

**Figure 5:**
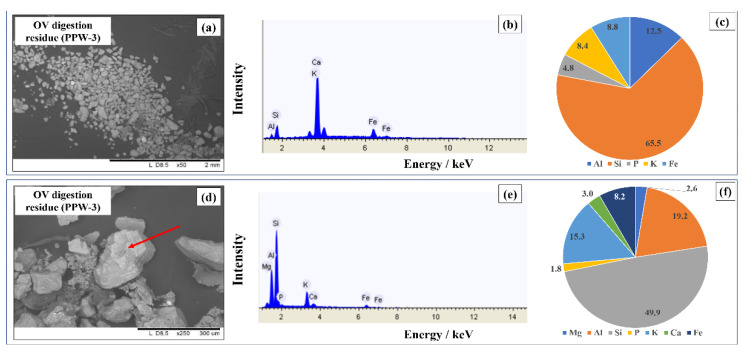
SEM-EDX analysis of the OV digestion procedure residue. (**a**) SEM image of the residue (4.0 mm × 3.0 mm, 50× magnification). (**b**) EDX spectrum of the area shown in (**a**). (**c**) Elemental composition of the residue. (**d**) SEM image of a particle of the residue (0.5 mm × 0.7 mm, 250× magnification). (**e**) EDX spectrum of the particle indicated in (**d**). (**f**) Elemental composition of the particle indicated in (**d**).

Student’s paired *t*-test was carried out in order to compare the extraction efficiency of the two methods (*p* = 0.05). The results of the test (t = 0.242 < 2.069) show that it is not possible to state that there is a statistical difference between the concentrations of the metals analysed with two methods of digestion. Despite these findings, it was interesting to note that, following the open vessel digestion of sample PPW-3, an insoluble residue remained at the bottom of the container. This deposit appeared dark grey in colour with black and brownish tips, mainly in the form of small particles. The residue was recovered with a minimal amount of Milli-Q water, filtered and analysed using scanning electron microscopy (Figure 5).

The size of the particles ranged from 50 μm to 300 μm and the spectroscopic analysis revealed the presence of Si, Al, K, P and Fe, accounting for the lower concentrations obtained in ICP-OES analysis with the OV procedure, as observed by a direct comparison of the values. Moreover, through punctual analysis it was possible to obtain a more complete chemical characterization of the particles (Figure 5d–f). Based on the results obtained, the composition of the residue is in accordance with the one of silica sands [31]. First of all, this substrate is only soluble in hydrofluoric acid and could not be dissolved using a 1:10 mixture of perchloric acid and nitric acid by heating to 120 °C. In addition, silica sands are composed mainly of SiO_2_, Al_2_O_3_ and Fe_2_O_3_, with the presence of other metallic oxides. So, it is possible that the PPW-3 sample was fortified with sands to increase the weight for the same number of pistils used.

## 4. Discussion

As demonstrated in this work, saffron quality can be assessed using a wide array of analytical techniques. Limiting this assessment to the UV-VIS spectrophotometric analysis described in the ISO 3632 technical standard can be misleading in some cases and can give an incomplete characterization of the sample. This work shows that with the aid of ATR-FTIR spectroscopy, along with SEM-EDX and ICP-OES analysis, it is possible to obtain a clearer picture of saffron quality. In this regard, morphological characterization and elemental composition analysis of saffron emerged as two promising and innovative factors for the analysis of this spice.

In fact, most of the samples studied showed little to no signs of adulteration or contamination despite being denied classification in one of the three commercial categories. This was especially the case for saffron filament samples, which all appeared similar in terms of morphological appearance, with no floral contamination, being homogenous in terms of elemental composition and with a metallic content in line with literature values. With the exception of PFL-3, all of these samples were denied classification solely due to the odorous power being outside the specified range, whereas the coloring and bittering power values were at least above the minimum requirements for classification in category III and the moisture and volatile matter content was below the ISO thresholds, indicating no signs of adulteration aimed at increasing saffron mass by addition of volatile substances. This confirms the previously mentioned findings of other studies which have demonstrated the inability of the ISO method in establishing saffron aroma [1,19] and the inadequacy of the odorous power as a parameter assessing the quality of the spice [21].

This was true also for saffron powders: if we do not consider the results obtained for the odorous power, samples characterized by coloring and bittering powers above the minimum requirement of category III and moisture and volatile matter content below 10% also showed a good quality when assessed with the other techniques. However, samples with a coloring and/or bittering power below the minimum value for classification in category III also showed a lower quality according to the other analyses. Specifically, these samples showed a higher content of all terrigenous elements (Al, Si, Na, Mg, Ca and Fe), their morphological appearance differed from the typical features of saffron, and in some cases floral contaminants were identified.

**Figure 6:**
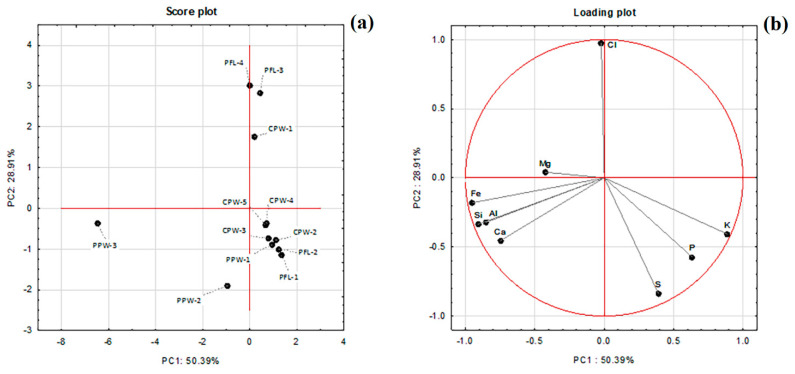
Results of the PCA statistical analysis. (**a**) Score plot. (**b**) Loading plot.

With the aim of trying to highlight some differences between saffron samples, a multivariate data analysis technique i.e., principal components analysis (PCA), was applied to the results of the EDX aerial analyses presented in Table 2. The loading plot and the score plot are reported in Figure 6. In spite of the limited number of samples, it seemed interesting to us to apply this approach, which in fact allowed us to draw some useful preliminary indications.

These results show that the majority of the samples are distinguished by the presence of potassium, phosphorous and sulphur, three elements which occur naturally in saffron and are essential for plant growth [32], and that are not generally related to sources of contamination. The correlation identified between these elements is in our opinion a confirmation of the validity of the approach. However, samples PFL-3, PFL-4 and CPW-1 differed from the others in their high chlorine content, which is the element with the highest loading on the second component. Finally, the samples of lower quality (PPW-2, PPW-3) correlate with terrigenous elements such as Fe, Al, Si, Ca, and Mg.

This was particularly evident for sample PPW-3, which is the one of low quality according to all the analyses performed. This sample was also singled-out by the results of the ICP-OES analysis. In fact, this was the only sample in which the values obtained were consistently above the literature values for almost all of the analysed metals, suggesting that metal content can also be used as a way to discriminate between high- and low-quality samples. In this regard, it is interesting to underline the complementarity of the two novel techniques employed in the determination of saffron quality, in addition to the fact that elemental composition and metal content revealed to be two important parameters in the spice’s evaluation. Indeed, both the absolute amounts (determined via ICP-OES) and the relative percentages (determined via SEM-EDX) can be used to distinguish between the quality of samples. 

## 5. Conclusions

This study shows that a multi-analytical approach is necessary in order to achieve a complete quality characterization of saffron. Each individual technique can give useful information, but it is only by considering the results of all the techniques employed that a clear picture of sample quality can be obtained. Indeed, the ISO 3632 UV-Vis spectrophotometric method gives useful information with regards to the “colouring” and “bittering” power of saffron. Moving on, FTIR spectra represent a fingerprint of the spice and can be useful as an initial characterization tool. Morphological characterization can be carried out through scanning electron microscopy, whereas elemental composition, which yields important information regarding the treatment quality and the presence of contaminants, can be assessed with an EDX microprobe. Finally, both open vessel and microwave-assisted digestion procedures can be used for sample preparation in ICP-OES analysis for the determination of metallic content, which is another important parameter to be considered when assessing saffron quality.

## Figures and Tables

**Table 1:** Commercial categories assessing the quality of saffron.

Commercial Category	Colouring Power	Bittering Power	Odorous Power
I	≥200	≥70	20–50
II	170–199	55–69	20–50
III	120–169	40–54	20–50

**Table 2:** Type and origin of saffron samples analyzed.

Sample	Type	Origin
PPW-1	Powder	Private, Iran
PPW-2	Powder	Private, Iran
PPW-3	Powder	Private, Iran
PPW-4	Powder	Private, Iran
PFL-1	Filaments	Private, Iran
PFL-2	Filaments	Private, Iran
PFL-3	Filaments	Private, Genova (Italy)
PFL-4	Filaments	Private, Genova (Italy)
PFL-5	Filaments	Private, Iran
PFL-6	Filaments	Private, Iran
PFL-7	Filaments	Private, Iran
PFL-8	Filaments	Private, Iran
PFL-9	Filaments	Private, Iran
PFL-10	Filaments	Private, Iran
PFL-11	Filaments	Private, Iran
PFL-12	Filaments	Private, Iran
CPW-1	Powder	Commercial, Italy
CPW-2	Powder	Commercial, Italy
CPW-3	Powder	Commercial, Italy
CPW-4	Powder	Commercial, Italy
CPW-5	Powder	Commercial, Italy

**Table 3:** Moisture and volatile matter content in saffron samples.

Sample	Moisture and Volatile Matter Content/%	s*/±%
PPW-1	4.524	0.001
PPW-2	5.582	0.001
PPW-3	5.722	0.001
PPW-4	6.287	0.001
PFL-1	4.126	0.001
PFL-2	4.156	0.001
PFL-3	11.653	0.005
PFL-4	4.914	0.005
PFL-5	9.069	0.001
PFL-6	9.543	0.001
PFL-7	7.825	0.001
PFL-8	6.072	0.001
PFL-9	8.355	0.001
PFL-10	8.399	0.001
PFL-11	7.609	0.001
PFL-12	5.913	0.001
CPW-1	9.324	0.004
CPW-2	6.425	0.003
CPW-3	7.480	0.003
CPW-4	4.833	0.006
CPW-5	8.752	0.003

* Standard deviation.

**Table 4:** Elemental composition of the saffron samples expressed as percentage by weight of the element’s oxide.

Sample	K		Mg		Al		Si		P		Ca		Cl		S		Fe	
	%wt	s *	%wt	s	%wt	s	%wt	s	%wt	s	%wt	s	%wt	s	%wt	s	%wt	s
PPW-1	68.6	3.5	1.9	1.5	2.0	1.7	2.9	0.6	8.0	1.5	6.6	2.1	1.7	2.0	5.3	0.7	3.1	1.3
PPW-2	56.5	6.0	2.2	1.4	5.0	2.9	7.3	1.4	7.9	1.5	9.8	3.9	2.7	1.3	6.0	1.4	2.9	1.6
PPW-3	30.6	2.6	2.7	0.4	6.3	2.4	14.5	2.9	4.3	1.2	16.7	2.5	6.4	2.5	4.1	0.8	14.4	3.8
PFL-1	66.7	5.6	1.3	1.4	0.4	0.7	4.3	1.6	7.8	1.5	9.2	4.3	4.4	2.0	5.9	1.5	<LOD	-
PFL-2	67.6	3.1	0.9	1.3	1.6	1.6	5.2	0.9	8.5	1.5	7.8	3.2	3.1	1.5	5.2	0.9	<LOD	-
PFL-3	54.5	3.1	1.9	0.8	1.6	0.8	2.6	1.2	5.9	1.1	3.2	1.7	26.4	0.9	3.8	0.8	<LOD	-
PFL-4	48.8	4.2	1.6	0.8	0.6	0.8	2.7	1.2	4.8	0.6	6.5	2.1	30.7	2.5	4.2	1.0	<LOD	-
CPW-1	53.3	1.8	2.3	0.5	0.9	1.0	2.4	0.8	7.2	1.0	8.0	0.9	21.2	2.2	4.0	0.9	1.1	0.6
CPW-2	70.0	1.7	1.6	0.5	1.0	1.2	3.1	1.0	6.7	1.1	9.2	2.5	2.9	0.8	5.6	0.4	<LOD	-
CPW-3	65.0	3.2	2.9	0.4	1.1	1.0	2.8	0.8	8.3	1.0	9.0	3.9	4.6	2.0	5.3	2.1	1.4	0.9
CPW-4	68.6	3.0	0.0	0.0	1.2	2.5	4.8	0.9	6.1	0.4	10.6	3.0	3.8	1.0	4.9	0.9	<LOD	-
CPW-5	66.2	5.3	2.4	0.7	2.0	1.9	3.8	1.1	7.7	0.9	6.0	0.8	4.7	2.4	5.1	1.1	2.5	2.0

* Standard deviation.

**Table 5:** Metal content in saffron samples determined using the open vessel (OV) procedure. Results are expressed in μg g^−1^.

Sample	K		Mg		Al		Na		Cu		Ca		Zn		Cr		Fe		Mn		Pb		Ni		**Cd**	
	C	s *	C	s	C	s	C	s	C	s	C	s	C	s	C	s	C	s	C	s	C	s	C	**s**	**C**	**s**
PFL-1	6840	30	780	30	24	1	43	6	8	1	347	9	27	1	<LOQ	-	24	1	<LOQ	-	<LOQ	-	<LOQ	-	<LOQ	-
CPW-5	6800	100	810	20	56	1	79	4	6	1	695	3	23	1	<LOQ	-	82	2	<LOQ	-	<LOQ	-	<LOQ	-	<LOQ	-
PPW-3	6600	100	2030	90	524	3	1250	60	43	1	6000	100	84	2	<LOQ	-	436	6	<LOQ	-	<LOQ	-	<LOQ	-	<LOQ	-

* Standard deviation.

**Table 6:** Metal content in saffron samples determined using the microwave digestion (MW) procedure. Results are expressed in μg g^−1^.

Sample	K		Mg		Al		Na		Cu		Ca		Zn		Cr		Fe		Mn		Pb		Ni		**Cd**	
	C	s *	C	s	C	s	C	s	C	s	C	s	C	s	C	s	C	s	C	s	C	s	C	**s**	**C**	**s**
PFL-1	5990	40	800	100	33	1	28	1	5	1	384	4	30	1	<LOQ	-	38	1	<LOQ	-	<LOQ	-	<LOQ	-	<LOQ	-
CPW-5	6670	70	1080	50	175	1	77	3	6	1	1035	5	25	1	<LOQ	-	254	7	<LOQ	-	<LOQ	-	<LOQ	-	<LOQ	-
PPW-3	6500	100	2000	100	916	5	293	8	47	1	6649	8	65	1	<LOQ	-	1477	7	<LOQ	-	<LOQ	-	<LOQ	-	<LOQ	-

* Standard deviation.

## Data Availability

All of the data collected in this study can be found within the article.

## References

[1] Sereshti H., Ataolahi S., Aliakbarzadeh G., Zarre S., Poursorkh Z. (2018). Evaluation of Storage Time Effect on Saffron Chemical Profile Using Gas Chromatography and Spectrophotometry Techniques Coupled with Chemometrics. J. Food Sci. Technol..

[2] Interlandi S. (2010). Aspetti Agronomici Innovativi dello Zafferano (*Crocus sativus* L.) in Sicilia. Ph.D. Thesis.

[3] Khilare V., Tiknaik A., Prakash B., Ughade B., Korhale G., Nalage D., Ahmed N., Khedkar C., Khedkar G. (2019). Multiple Tests on Saffron Find New Adulterant Materials and Reveal That Ist Grade Saffron Is Rare in the Market. Food Chem..

[4] Domenighini M. (2014). La Coltivazione dello Zafferano in Valle Camonica. Master’s Thesis.

[5] Schweiggert R.M. (2018). Perspective on the Ongoing Replacement of Artificial and Animal-Based Dyes with Alternative Natural Pigments in Foods and Beverages. J. Agric. Food Chem..

[6] Alighaleh P., Khosravi H., Rohani A., Saeidirad M.H., Einafshar S. (2022). The Detection of Saffron Adulterants Using a Deep Neural Network Approach Based on RGB Images Taken under Uncontrolled Conditions. Expert Syst. Appl..

[7] Kuchta K., Aritake K., Urade Y., Tung N.H., Yuan C.-S., Sasaki Y., Shimizu K., Shoyama Y. (2022). Preventing Dementia Using Saffron, The Kampo Medicine, Kamiuntanto, and Their Combination, Kamiuntantokabankoka. Front. Pharmacol..

[8] José Bagur M., Alonso Salinas G.L., Jiménez-Monreal A.M., Chaouqi S., Llorens S., Martínez-Tomé M., Alonso G.L. (2017). Saffron: An Old Medicinal Plant and a Potential Novel Functional Food. Molecules.

[9] Siddiqui S.A., Ali Redha A., Snoeck E.R., Singh S., Simal-Gandara J., Ibrahim S.A., Jafari S.M. (2022). Anti-Depressant Properties of Crocin Molecules in Saffron. Molecules.

[10] Lian J., Zhong Y., Li H., Yang S., Wang J., Li X., Zhou X., Chen G. (2022). Effects of Saffron Supplementation on Improving Sleep Quality: A Meta-Analysis of Randomized Controlled Trials. Sleep Med..

[11] Marx W., Lane M., Rocks T., Ruusunen A., Loughman A., Lopresti A., Marshall S., Berk M., Jacka F., Dean O.M. (2019). Effect of Saffron Supplementation on Symptoms of Depression and Anxiety: A Systematic Review and Meta-Analysis. Nutr. Rev..

[12] Ferrari S. (2011). Studio Preliminare Sulle Caratteristiche Qualitative dello Zafferano in Valle Camonica. Master’s Thesis.

[13] Alonso G.L., Salinas M.R., Sànchez-Fernàndez M.A., Garijo J. (2000). Note. Physical parameters in controlling saffron quality. Food Sci. Tech. Int..

[14] Soffritti G., Busconi M., Sánchez R.A., Thiercelin J.M., Polissiou M., Roldán M., Fernández J.A. (2016). Genetic and Epigenetic Approaches for the Possible Detection of Adulteration and Auto-Adulteration in Saffron (*Crocus Sativus* L.) Spice. Molecules.

[15] Petrakis E.A., Polissiou M.G. (2017). Assessing Saffron (*Crocus Sativus* L.) Adulteration with Plant-Derived Adulterants by Diffuse Reflectance Infrared Fourier Transform Spectroscopy Coupled with Chemometrics. Talanta.

[16] De Benedettis P. (2013). Aspetti Quanti-Qualitativi della Produzione di Zafferano in Brianza: Confronto tra Bulbi di Differenti Aree Geografiche. Bachelor’s Thesis.

[17] (2011). Spices—Saffron (*Crocus sativus* L.).

[18] (2010). Spices—Saffron (*Crocus sativus* L.).

[19] Bononi M., Milella P., Tateo F. (2015). Gas Chromatography of Safranal as Preferable Method for the Commercial Grading of Saffron (*Crocus Sativus* L.). Food Chem..

[20] Valle García-Rodríguez M., Serrano-Díaz J., Tarantilis P.A., López-Córcoles H., Carmona M., Alonso G.L. (2014). Determination of Saffron Quality by High-Performance Liquid Chromatography. J. Agric. Food Chem..

[21] Hadizadeh F., Mahdavi M., Emami S.A., Khashayarmanesh Z., Hassanzadeh M., Asili J. (2007). Evaluation of ISO Method in Saffron Qualification. Acta Hortic..

[22] Kiani S., Minaei S., Ghasemi-Varnamkhasti M. (2018). Instrumental Approaches and Innovative Systems for Saffron Quality Assessment. J. Food Eng..

[23] Ordoudi S.A., Cagliani L.R., Melidou D., Tsimidou M.Z., Consonni R. (2017). Uncovering a Challenging Case of Adulterated Commercial Saffron. Food Control.

[24] Ordoudi S.A., de Los Mozos Pascual M., Tsimidou M.Z. (2014). On the Quality Control of Traded Saffron by Means of Transmission Fourier-Transform Mid-Infrared (FT-MIR) Spectroscopy and Chemometrics. Food Chem..

[25] Jia L.H., Liu Y., Li Y.Z. (2011). Determination of the Major Metal Elements Including Heavy Metals in Saffron from Tibet and Henan by ICPAES or ICPMS. J. Chin. Pharm. Sci..

[26] D’Archivio A.A., Giannitto A., Incani A., Nisi S. (2014). Analysis of the Mineral Composition of Italian Saffron by ICP-MS and Classification of Geographical Origin. Food Chem..

[27] Rodushkin I., Ruth T., Huhtasaari A. (1999). Comparison of Two Digestion Methods for Elemental Determinations in Plant Material by ICP Techniques. Anal. Chim. Acta.

[28] Varliklioz Er S., Eksi-Kocak H., Yetim H., Boyaci I.H. (2017). Novel Spectroscopic Method for Determination and Quantification of Saffron Adulteration. Food Anal. Methods.

[29] Caiola M.G., Di Somma D., Lauretti P. (2000). Comparative Study of Pollen and Pistil in *Crocus Sativus* L. (Iridaceae) and Allied Species. Ann. Bot..

[30] Xu X., Du X., Wang F., Sha J., Chen Q., Tian G., Zhu Z., Ge S., Jiang Y. (2020). Effects of Potassium Levels on Plant Growth, Accumulation and Distribution of Carbon, and Nitrate Metabolism in Apple Dwarf Rootstock Seedlings. Front. Plant Sci..

[31] Casali De Rosa V. (2019). Process Simulation of a Mineral Processing Plant: Water Balance for Environmental Sustainability. Master’s Thesis.

[32] Ruan J., Wu X., Ye Y., Hardter R. (1998). Effect of Potassium, Magnesium and Sulphur Applied in Different Forms of Fertilisers on Free Amino Acid Content in Leaves of Tea (*Camelia sinensin* L.). J. Sci. Food Agric..

